# Monographic Quality Parameters and Genotoxicity Assessment of *Asphodelus bento-rainhae* and *Asphodelus macrocarpus* Root Tubers as Herbal Medicines

**DOI:** 10.3390/plants11223173

**Published:** 2022-11-20

**Authors:** Maryam Malmir, Rita Serrano, Katelene Lima, Maria Paula Duarte, Isabel Moreira da Silva, Beatriz Silva Lima, Manuela Caniça, Olga Silva

**Affiliations:** 1 Research Institute for Medicines (iMed.ULisboa), Faculty of Pharmacy, Universidade de Lisboa, 1649-003 Lisbon, Portugal; 2 MEtRICs/Chemical Department, Nova School of Science and Technology, Universidade Nova de Lisboa, 2829-516 Caparica, Portugal; 3 National Reference Laboratory of Antibiotic Resistances and Healthcare-Associated Infections, Department of Infectious Diseases, National Institute of Health Dr. Ricardo Jorge, 1649-016 Lisbon, Portugal

**Keywords:** *Asphodelus bento-rainhae*, *Asphodelus macrocarpus*, herbal medicines, quality control, root tubers

## Abstract

Root tubers of *Asphodelus bento-rainhae* subsp. *bento-rainhae* (AbR), an endemic species with relevant interest due to conservation concerns, and *Asphodelus macrocarpus* subsp. *macrocarpus* (AmR) have been traditionally used for culinary and medicinal purposes, mainly associated with skin infection and inflammation. The present study aims to establish the quality control criteria for the proper characterization of dried root tubers of both species as herbal substances, together with their preclinical safety assessments. Botanical identification using macroscopic and microscopic techniques and phytochemical evaluation/quantification of the main classes of marker secondary metabolites, including phenolic compounds (flavonoid, anthraquinone, condensed and hydrolysable tannin) and terpenoids were performed. Additionally, in vitro genotoxicity/mutagenicity was evaluated by Ames test. Evident morphological differences in the development of tubercles (3.5 × 1 cm in AbR and 8.7 × 1.4 cm in AmR) and microscopicly in the arrangements and characteristics of the vascular cylinder (metaxylem and protoxylems) were found. Anatomical similarities such as multiple-layered epidermis (velamen) and the cortex area with thin-walled idioblasts (134 ± 2.9 µm and 150 ± 27.6 µm) containing raphide crystals (37.2 ± 14.2 µm and 87.7 ± 15.3 µm) were observed between AbR and AmR, respectively. Terpenoids (173.88 ± 29.82 and 180.55 ± 10.57 mg OAE/g dried weight) and condensed tannins (128.64 ± 14.05 and 108.35 ± 20.37 mg CAE/g dried weight) were found to be the main class of marker secondary metabolites of AbR and AmR extracts, respectively. No genotoxicity (up to 5 mg/plate, without metabolic activation) was detected in these medicinal plants’ tested extracts. The obtained results will contribute to the knowledge of the value of the Portuguese flora and their future commercial cultivation utilization as raw materials for industrial and pharmaceutical use.

## 1. Introduction

Medicinal plants and herbal medicines provide effective and affordable primary health care for much of the world’s population. Therapies involving these agents, with a long history of traditional use in several cultures, still offer great potential in the treatment of various diseases; however, they do not comprise an adequate assessment of their authenticity, quality, and safety. In fact, to date, many of them remain unproven and rarely monitored [1,2]. The genus *Asphodelus* L. belonging to the family *Asphodelaceae* is among the most popular source of medicinal plants in the Iberian Peninsula [3]. Root tubers of *Asphodelus* species have shown to be the most common plant part traditionally used for the treatment of skin-related disorders and infections such as wounds, eczema, alopecia, and psoriasis [4]. Besides the medicinal uses, tubers have been used as daily food after being moistened and fried beforehand to eliminate the astringent compounds [3,5]. As already stated by us [4], the local people of Iran, Turkey and Egypt use the root tubers of *A. aestivus* and *A. microcarpus* to produce a strong glue used by shoemakers and cobblers [3,6,7] and as yellow and brown dyes to dye wool [3].

A broad range of in vitro and in vivo biological activities of *Asphodelus* species root extracts have been documented [4] and found to have antimicrobial [8,9,10,11,12,13,14], antiparasitic [15], antimalarial [16], antitumoral [17,18,19], antioxidant [17,20,21,22,23], anti-inflammatory [24,25,26,27], hypotensive and diuretic [28] activities. They were mainly reported to have anthraquinone derivatives, triterpenoids, and naphthalene derivatives as major secondary metabolites [13,15,16,29,30,31,32,33,34,35].

According to the *Flora Iberica* [3], 12 *Asphodelus* species are present in the Iberian region, and based on the information obtained from the World Checklist of Selected Plant Families [36], the Checklist of the Vascular Plants of Portugal (Checklist da Flora de Portugal) [37], and Flora-On [38], only eight, namely, *Asphodelus aestivus* Brotero, *Asphodelus bento-rainhae* P. Silva (subsp. *bento-rainhae*), *Asphodelus fistulosus* Linnaeus (subsp. *fistulosus*; subsp. *madeirensis* Simon), *Asphodelus lusitanicus* Coutinho (var. *lusitanicus*; var. *ovoideus* (Merino) Z. Díaz and Valdés), *Asphodelus macrocarpus* Parlatore (subsp. *macrocarpus*; var. *arrondeaui* (J. Lloyd) Z. Díaz and Valdés) *Asphodelus ramosus* Linnaeus (subsp. *distalis* Z. Díaz and Valdés; subsp. *ramosus*), *Asphodelus serotinus* Wolley-Dod and *Asphodelus tenuifolius* Cavanilles, exist in Portugal with high distribution within the mainland and Madeira islands.

Among the species mentioned above, *Asphodelus bento-rainhae* subsp. *bento-rainhae* is an endemic species from the Gardunha mountain range “Serra da Gardunha,” located in the central region of Portugal, and considered vulnerable on the International Union for the Conservation of Nature (IUCN) Red List of Threatened Species [39]. It is located only in this region, covering the counties of Fundão and Castelo Branco [38], coexisting with *Asphodelus macrocarpus* subsp. *macrocarpus* in the same geographical area. They are known by the common Portuguese name *abrotea*, and their root tubers have been traditionally used for the treatment of scabies, dermatophytosis and warts in Portugal. General botanical and systematic descriptions of these species have been discussed by several taxonomists in various flora publications [3,40,41,42], describing them as perennial and glabrous herbs with horizontal or oblique rhizomes (with some or any fibrous remains of old leaves) and short, thick, fusiform root tubercles, more abruptly narrowed in the distal part than in the proximal [43,44].

Although there are ethnomedical data supported by botanical, phytochemical and biological studies of *Asphodelus* species, to the best of our knowledge, no scientific studies have been documented on *Asphodelus bento-rainhae* and *Asphodelus macrocarpus*. Moreover, considering their threatened status and valuable traditional and medicinal properties, their conservation and further commercial cultivation are extremely important. Therefore, the present study was conducted to establish the principal botanical and chemical specifications of their root tubers following the official quality monograph criteria, together with a preclinical safety assessment of both species to allow their future use as herbal substances for human use.

## 2. Results and Discussion

### 2.1. Botanical Characterization

Although macroscopic and microscopic observations of flowers [45,46,47,48,49,50,51,52,53,54] and leaves [55] of several *Asphodelus* species have been documented, data regarding the anatomy of root tubers are scarce.

**Figure 1:**
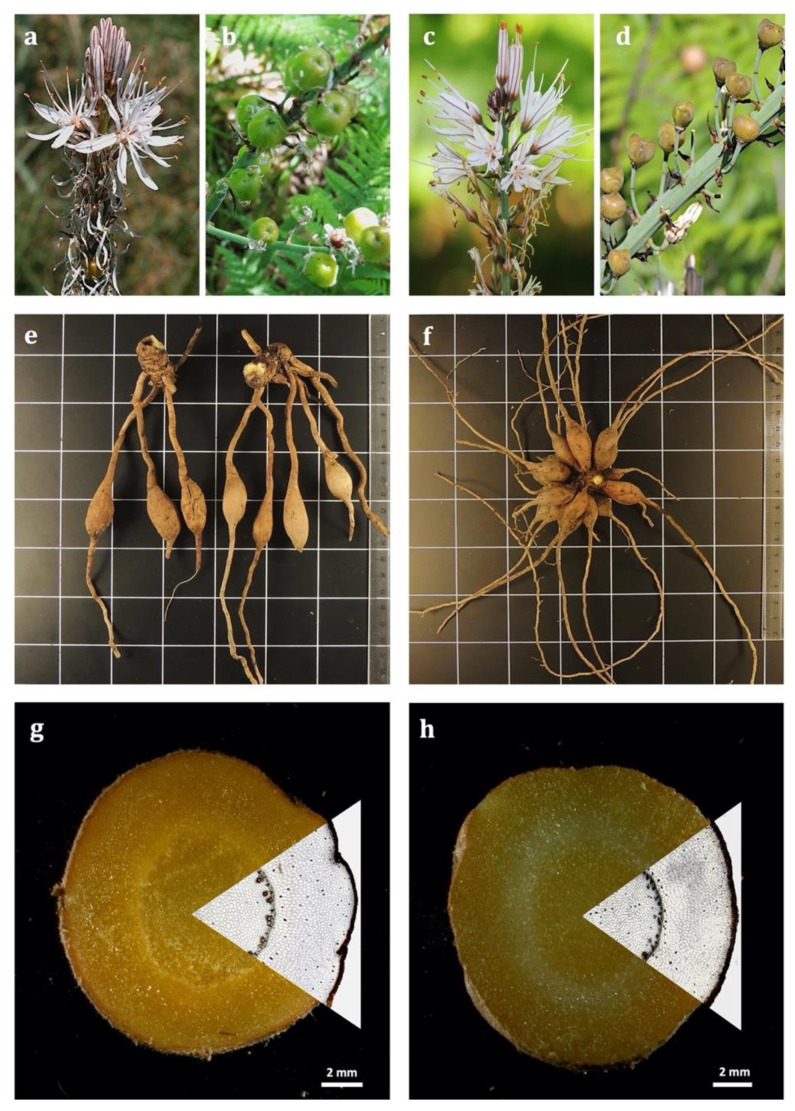
Macroscopic features of Portuguese *Asphodelus* species. Images of flowers and fruits of *A. macrocarpus* (**a**,**b**) and *A. bento-rainhae* (**c**,**d**) growing in their natural habitats, Serra da Gardunha, Fundão. Root tubers in general and cross-section views in *A. macrocarpus* (**e**,**g**) and *A. bento-rainhae* (**f**,**h**).

**Figure 2:**
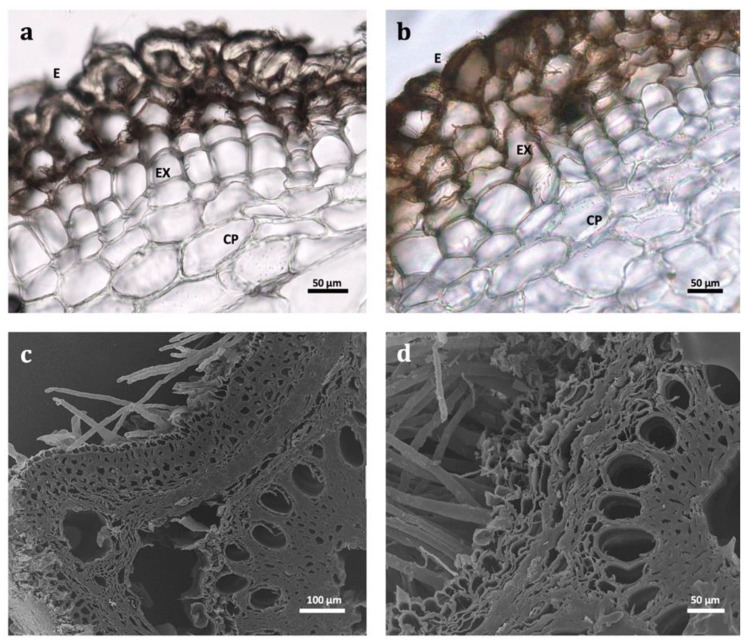

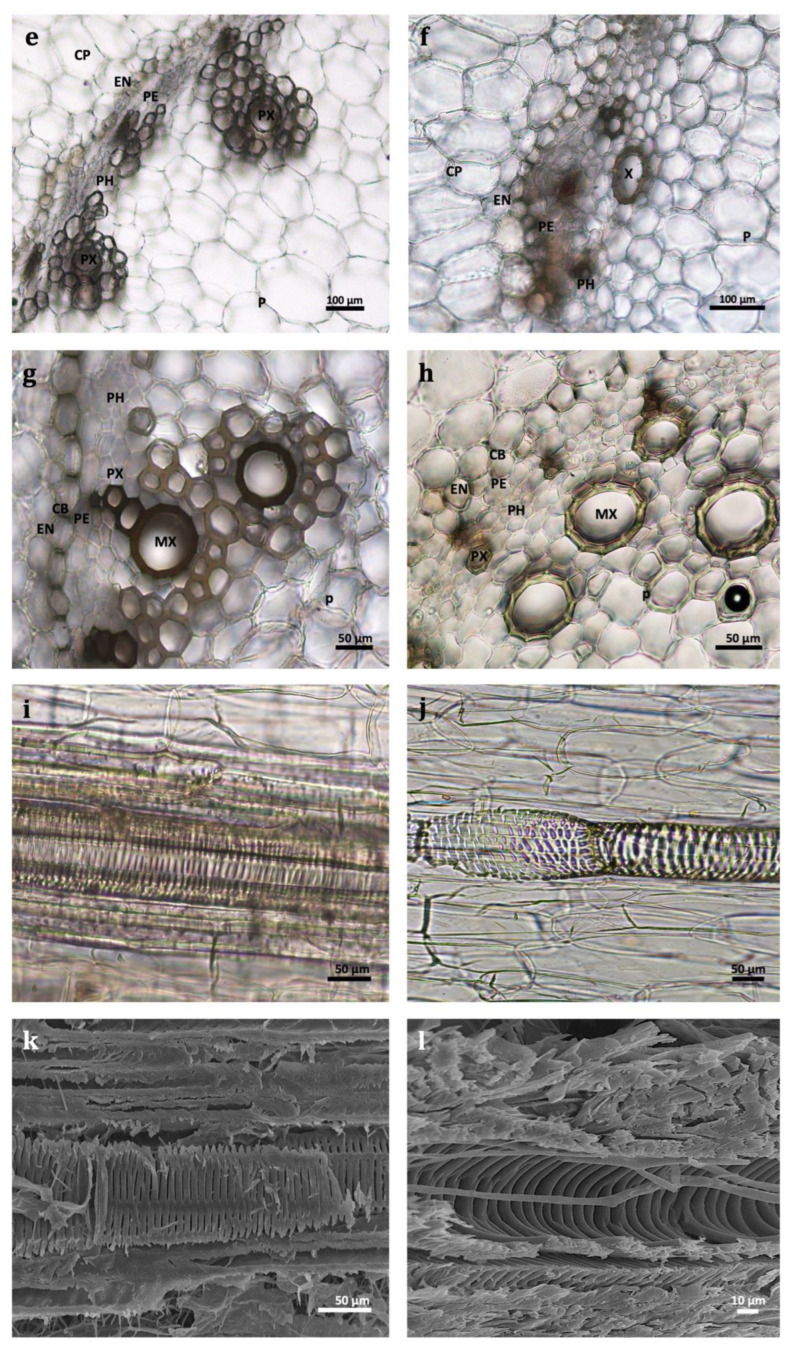

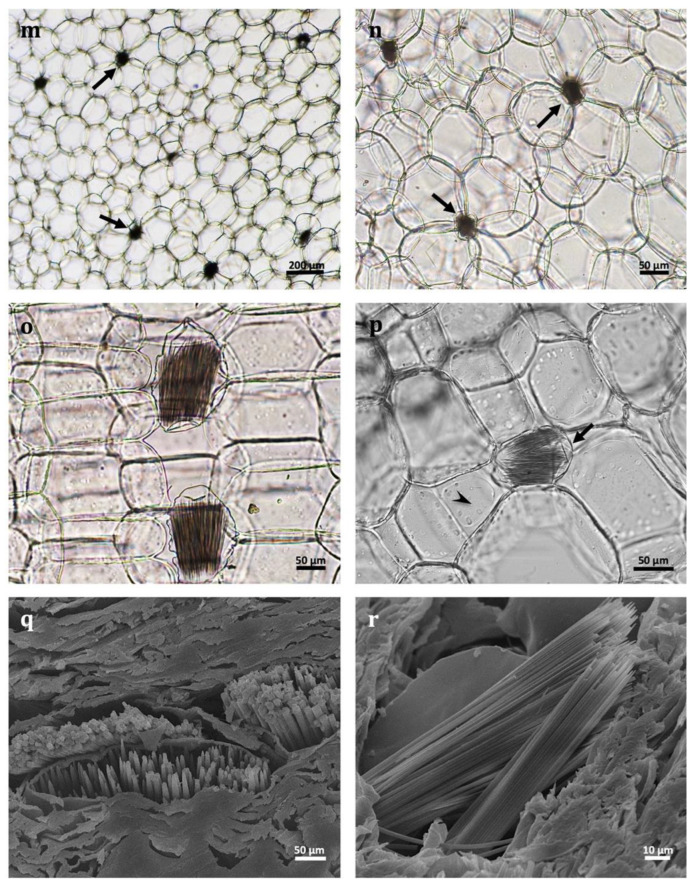
Microscopic features of Portuguese *Asphodelus* species. LM (**a**,**b**,**e**,**f**,**g**,**h**) and SEM (**c**,**d**) images of velamen with multiple layers (E—epidermis, EX—exodermis cells with suberized walls, CP—cortical parenchyma cells) in *A. macrocarpus* (**a**,**c**) and *A. bento-rainhae* (**b**,**d**) together with root hairs. Details of cortical parenchyma cells (CP), endodermis (EN), Casparian bands (CB) and vascular cylinder (PE—pericycle; PX—protoxylem; MX—metaxylem; PH—phloem; P—pith cells) in *A. macrocarpus* (**c**,**e**,**g**) and *A. bento-rainhae* (**d**,**f**,**h**) in transversal view. LM (**i**,**j**) and SEM (**k**,**l**) images of scalariform vessels with lignified secondary cell wall deposition and fibers in longitudinal view in *A. macrocarpus* (**i**,**k**) and *A. bento-rainhae* (**j**,**l**). LM (**m**–**p**) and SEM (**q**,**r**) images of cortex parenchyma showing thin-walled idioblasts with numerous calcium oxalate crystals, type raphides (arrows) in transversal view (**m**,**n**,**q**) and longitudinal view (**o**,**p**,**r**) in *A. macrocarpus* (**m**,**o**,**q**) and *A. bento-rainhae* (**n**,**p**,**r**); Details of parenchyma cells with polysaccharides (**p**) such as starch grains (arrowhead).

Considering our obtained data, macroscopically (Figure 1, Table 1), morphological variations were observed between the studied species in accordance with the general botanical description found in the *Flora Iberica*. The root tubers of *A. bento-rainhae* were short, fusiform, more abruptly narrowed in the distal part than in the proximal (2–5 cm × 0.7–1.6 cm) and developed directly on the rhizome, up to 2 cm; however, in *A. macrocarpus* (6–13 cm × 1.2–1.7 cm) they developed at a distance of 2–7 cm from the rhizome. Microscopicly (Figure 2, Table 1), the multiple-layered epidermis (velamen), without cuticle on the outer surface (Figure 2a–d, Table 1) and an average of 4–5 cells wide in both species. In addition, single-celled hairs (Figure 2c,d), which are responsible for rapid water uptake, water loss reduction, osmotic and mechanical protection, were observed.

The cortex area (Figure 2e,f, Table 1) between the velamen and central cylinder is up to ±24 cells wide in *A. bento-rainhae* and ±37 in *A. macrocarpus*, composed of oil cells and thin-walled idioblasts (58.3–62.5 μm in AbR and 60.7–114.6 μm in AmR). They contain numerous needle-shaped (raphides) crystals of calcium oxalate (20.8–62.5 μm in AbR and 78–114.3 μm in AmR) in their vacuole (Figure 2m–r, Table 1). The uniseriate endodermis cells (Figure 2e–h, Table 1) with Casparian strips, are periclinally orientated with thick walls. The vascular cylinder comprises the uniseriate pericycle, periclinally orientated (Figure 2e–h), and isodiametric cells. The root xylem (protoxylem and metaxylem) consists of vessels in short radial rows (Figure 2g–j, Table 1), alternating with broadly elliptical to variable-shaped clusters of phloem cells (Figure 2e–h). The vascular tissue is surrounded by sharply differentiated, somewhat thick-walled, polygonal parenchyma. The parenchymatous pith (Figure 2c–h, Table 1) comprises oval and almost circular, thin-walled cells with triangular, square, and rectangular intercellular spaces. Considering the abovementioned common anatomical structures between the two species, a noticeable size difference is evident. The width of idioblast cells and the length of raphides and endodermis cells are larger in *A. macrocarpus* compared to *A. bento-rainhae* (Table 1). The two species also differ considerably in the arrangements and characteristics of the vascular cylinder (metaxylem and protoxylems). Metaxylem (Figure 2e–h, Table 1) vessels in *A. macrocarpus* (±80 µm diameter) were found to be surrounded by several protoxylems; however, in *A. bento-rainhae* (±70 µm diameter), they are individually separated. These anatomical differences can be helpful in differentiating the two species in their dried whole, fragmented or powdered forms.

Multiseriate epidermis (velamen), enabling quick gain of transiently available soil water, the large parenchyma water-storing cells, the cortex cells containing soluble sugars, the oil cells containing lipid material of possibly defense character, and the cell idioblasts, which contain raphide crystals, were found among numerous anatomical similarities, between the root tubers of *A. aestivus*, our studied Portuguese *Asphodelus* and several other species from *Liliaceae* and *Orchidaceae*. These characteristics are, in fact, the means of synchronization and adaptation of these plants with the seasonality of the Mediterranean climate, as discussed by Sawidis et al., 2005 [56].

### 2.2. Phytochemical Analysis

Thin-layer chromatography (TLC) in this study was used as a rapid, reliable (due to its high sensitivity), and inexpensive technique for monitoring and detection of several samples, which could be analyzed simultaneously with low solvent usage [57,58]. Followed by the TLC method and for a more detailed phytochemical screening of the extracts and their constituents, high-performance liquid chromatography (HPLC) technique coupled to a photodiode detector (-UV/DAD) was applied.

**Figure 3:**
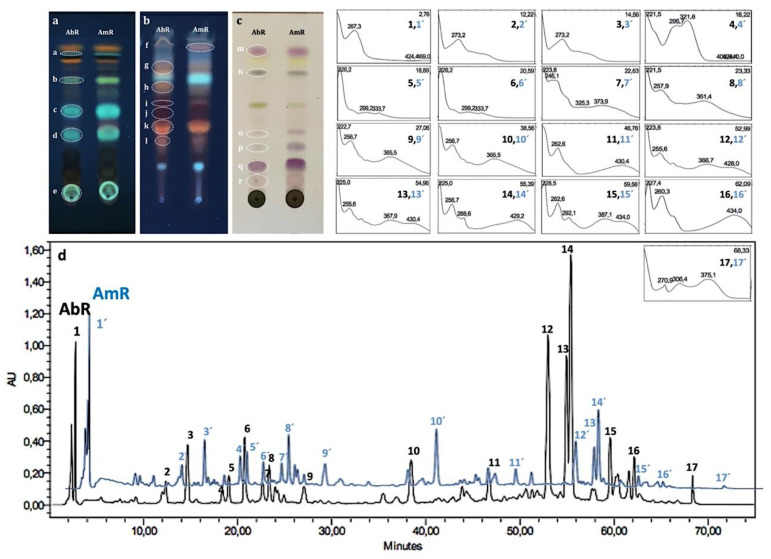
TLC and HPLC-UV/DAD chromatographic profiles of secondary metabolites of *A. bento-rainhae* and *A. macrocarpus* root tuber extracts. Phenolic acid derivatives detection, SiG60, spraying with NP/PEG, solvent system: S1, UV:366_nm_ (**a**); anthracene derivatives detection, SiG60 RP_18_, spraying with KOH 5%, solvent system: S2, UV:366_nm_ (**b**); terpenoids detection, SiG60 spraying with anisaldehyde-sulphuric acid, solvent system: S3, visible light (**c**); comparative chromatograms of AbR and AmR extracts (**d**).

The obtained TLC fingerprint (Figure 3a–c) confirmed the presence of phenolic acids (bands (a–e) with light to greenish blue colors under 366_nm_ UV light), anthracene derivatives (bands (f–l) with orangish to red color under 366_nm_ UV light) and terpenoids (bands (m–r) with pink to purple color, using an increased temperature under visible light) after spraying with specific revealing reagents in both species. The presence of phenolics and coumarins in *A. microcarpus* root extracts using TLC was previously reported by Abuhamdah et al., 2013 [10]. The HPLC-UV/DAD chromatographic profiles of both species (Figure 3d) were qualitatively similar in their chemical composition, characterized by the presence of phenolic acids and anthracene derivatives, based on spectral analysis, and compared with the data in the literature.

Considering the quantification results of the principal chemical classes of marker secondary metabolites (Table 2), TTC (173.88 ± 29.82, 180.55 ± 10.57 mg OAE/g dried weight) and TCTC (128.64 ± 14.05, 108.35 ± 20.37 mg CAE/g dried weight) were the main chemical classes of *A. bento-rainhae,* and *A. macrocarpus* extracts, respectively. Noticeably, anthraquinones exhibited the least content among all the other phenolic compounds in both species. However, in the studies related to the chemical composition of other *Asphodelus* species root extracts, they were referred to as the main chemical class responsible for their biological activities [4].

The statistical analysis of our results also showed that the contents of the marker secondary metabolites of AbR and AmR were dependent on the sample collection season. In fact, in AbR extracts, the total TAC, TCTC, and TFC content (3.21 ± 0.21 mg RhE/g dried weight, 128.64 ± 14.05 and 16.71 ± 1.12 mg CAE/g dried weight, respectively) were significantly higher in the second season collection in comparison with its first one (*p*-values: 0.011, 0.022 and 0.02, respectively), and for AmR extracts, the content of TAC (3.38 ± 0.26 ma mg RhE/g dried weight) was significantly higher in the first season collection than in the second season (*p*-value: 0.023). Concerning the chemical content of AmR and AbR extracts of the samples collected in the same season, results showed that TFC content (18.90 ± 0.26 mg CAE/g dried weight) in the first season was significantly higher in AmR when compared to AbR (*p*-value: 0.003).

The previously reported TPC and TFC values for other *Asphodelus* root extracts indicated the critical role of solvent selection in the extraction procedure. In fact, *A. microcarpus* ethanolic [20] (39.35 ± 4.2 mg GAE/g of dry weight) and methanolic [20,23] (15.31 ± 7.8 and 17.90 GAE/g of dry weight) extracts exhibited significantly higher amounts of total polyphenols in comparison to an aqueous extract [20]. However, Mayouf et al., 2019 [59] reported a significantly elevated amount of total polyphenols (377 ± 0.030 mg GAE/g of dry weight) in methanolic extracts of this species. Total flavonoids of methanolic extracts of *A. microcarpus* reported by Di Petrillo et al. [20] (3.94 ± 1.05 mg QUE/g of dry weight) were significantly smaller than the ones reported by Kitaz 2017 [23], (14.69 mg RUE/g of dry weight). Dichloromethane and ethyl acetate extracts of the roots of *A. albus* and *A. aestivus* showed high phenolic content (30.74 ± 0.41, 20.21 ± 0.19 mg GAE/g of dry weight, respectively) and *A. aestivus* presented higher flavonoid content (13.82 ± 0.80, mg RE/g of dry weight) when compared to *A. albus* [60]. Hydromethanolic extracts of *A. tenuifolius* [14] showed smaller amounts of polyphenols and flavonoids (11.4 ± 0.82 mg GAE/g of dry weight, 3.2 ± 0.08 QUE/g of dry weight, respectively) in comparison to our obtained contents of hydroethanolic extracts of both species. The presence of alkaloids, flavonoids, and tannins in *A. tenuifolius* root extracts using a colorimetric test tube was also previously reported by Menghani et al., 2012 without quantification data [12].

Overall and as discussed by Kitaz, 2017 [23], the difference in amounts of secondary metabolites in different *Asphodelus* species, is probably related to geographical, environmental, and climatic factors and conditions, processing methods, and other intrinsic (genetic, extracting solvent) and extrinsic (environmental, handling and development stage) factors.

### 2.3. Preclinical Safety Assessment

The Ames test has been used as an important tool in genetic toxicology for the assessment of chemical compounds’ safety due to the positive correlation between mutagenicity and carcinogenicity [61]. Furthermore, the presence of mutagenic compounds in plant extracts has been raising concerns about the carcinogenic risks resulting from the long-term use of plants as food, medicines, and source of raw materials in the pharmaceutical industry; therefore, genotoxicity studies (e.g., Ames test) are extremely important to assess the preclinical safety of plant extracts/herbal preparations to verify their mutagenic potential for both safety and economic purposes [62,63,64].

Following the guidelines on genotoxicity, for a substance to be considered genotoxic in the Ames test, the number of revertant colonies on the plates containing the test compounds/substance must be more than twice the number of colonies produced on the solvent control plates (i.e., a ratio above 2.0). In addition, a positive dose-response should be evident for the various concentrations of the tested mutagen [65,66]. Considering the obtained data of the quantification analysis of all the collected samples and their consecutive extracts, the obtained extracts from the second season collection (AbRb, AmRb), exhibited the higher contents of the main classes of secondary metabolite and subsequently were selected for further examination of their safety.

According to our obtained results presented in Table 3., in the plate assay method without metabolic activation, both AbRb and AmRb extracts did not induce an increase in the number of revertant colonies in any of the tested strains at any tested concentration (250, 625, 1250, 2500, 3750 and 5000 µg of extracts/plate). Cytotoxicity did not occur, since there was neither a decrease in the number of spontaneous revertants nor a decrease on the background lawn of the plates, in any of the concentrations tested. Therefore, under the conditions of this study, the extracts of both species did not show mutagenic activity, which is crucial to ensure their safety [61,66,67].

## 3. Materials and Methods

### 3.1. Reagents

Acetone (CH_3_COCH_3_), aluminum chloride (AlCl_3_), 9-aminoacridine hydrochloride monohydrate, ammonium sodium phosphate dibasic tetrahydrate (NaNH_4_HPO_4_ · 4H_2_O), *d*-(+)-biotin (C_10_H_16_N_2_O_3_S), dimethyl sulfoxide/DMSO [(CH_3_)_2_SO], gallic acid [C_6_H_2_(OH)_3_COOH], glucose monohydrate (C_6_H_12_O_6_.H_2_O), 2-nitrofluorene (C_13_H_9_NO_2_), *tert*-butyl hydroperoxide/T-BHP [(CH_3_)_3_COOH], and vanillin [4-(HO)C_6_H_3_-3-(OCH_3_)CHO], were obtained from Sigma-Aldrich (St. Louis, MO, USA). Ferric chloride hexahydrate (FeCl_3_.6H_2_O), hydrochloric acid (HCl), l-histidine monohydrochloride monohydrate (C_6_H_9_N_3_O_2_·HCl·H_2_O), magnesium sulfate heptahydrate (MgSO_4_.7H_2_O), methanol (CH_3_OH), perchloric acid (HClO_4_), potassium iodate (KIO_3_), sodium acetate trihydrate (CH_3_COONa.3H_2_O), sodium carbonate (Na_2_CO_3_), sodium hydroxide (NaOH), sodium nitrite (NaNO_2_), were purchased from Merck (Germany). (+)-Catechin (C_15_H_14_O_6_) and oleanolic acid (C_30_H_48_O_3_) were acquired from Extrasynthese (Genay, France). Citric acid monohydrate [HOC(COOH)(CH_2_COOH)_2_·H_2_O], di-sodium hydrogen phosphate dihydrate (Na_2_HPO_4_·2H_2_O), and sodium dihydrogen phosphate monohydrate (NaH_2_PO_4_ · H_2_O) were purchased from PanReac AppliChem (Barcelona, Spain). Sodium chloride (NaCl) and di-potassium hydrogen phosphate (K_2_HPO_4_) were from Honeywell Fluka™ (Seelze, Germany). Bacto™ agar from Becton Dickinson and Co (Franklin Lakes, NJ, USA), *n*-butanol [CH_3_(CH_2_)_3_OH] from Fisher Scientific^TM^ (Loughborough, UK), ferrous sulfate heptahydrate (FeSO_4_.7H_2_O) from MandB laboratory chemicals (London, UK), Folin-Ciocalteu from Biochem chemopharma (Cosne-Cours-sur-Loire, France), glacial acetic acid (CH_3_CO_2_H) from Chem-Lab NV (Zedelgem, Belgium), sodium azide from J.T. Baker Chemical Company ((Phillipsburg, NJ, USA) and nutrient broth (NB) nº 2 from Oxoid ((Basingstoke, UK) were acquired. In the preparation of all solutions, dilutions, and culture media, ultra-pure water from a Milli-Q water purification system, Millipore (Molsheim, France), was used.

### 3.2. Plant Material

#### 3.2.1. Sample Collection

Root tubers of *A. bento-rainhae* (AbR) and *A. macrocarpus* (AmR), were collected from Serra da Gardunha, Portugal, first at the early flowering stage (AbRa and AmRa) in May 2019 and then for the second time, during the root dormancy (AbRb and AmRb) in November 2019. Then, they were identified by Maria Cristina Duarte, scientific curator of the LISC Herbarium and plant collections in the Tropical Botanical Garden and Lisbon Botanical Garden. All samples were dried in a well-ventilated, dark space at room temperature. Corresponding voucher specimens were deposited in the Laboratory of Pharmacognosy, Department of Pharmacy, Pharmacology and Health Technologies, Faculty of Pharmacy, Universidade de Lisboa (voucher specimens’ number: OSilva_201901—*A. bento rainhae* and OSilva_201902—*A. macrocarpus*).

#### 3.2.2. Botanical Identification

Macroscopic and microscopic analyses of the dried root tubers, as raw material for medicine production, were performed according to the *Portuguese Pharmacopoeia* (9th edition, 2008). An Olympus SZ61 optical stereo microscope (Heerbrugg, Switzerland) coupled with an Olympus ColorView IIIu CCD 5.0MP camera featuring 2576 × 1932-pixel resolution (Tokyo, Japan) was used for macroscopic examination. For the light microscopy (LM), manually prepared sample sections were mounted in 60% aqueous chloral hydrate solution and examined using an Olympus CX40 upright microscope (York, UK), coupled with a Leica MC170 HD camera. Image analysis was performed with the Leica application suite (LAS) software, version 4.8.0. For Scanning Electron Microscopy (SEM), selected plant materials were sectioned, dehydrated at 35 °C for 24 h, and directly mounted on stubs using double-sided adhesive tape. Prepared samples were then sputtered with a thin layer of gold in a Polaron E 5350 and observed using a JEOL JSM-T220 scanning electron microscope at 15 kV, with a digital image acquisition integrated system (Peabody, MA, USA).

### 3.3. Preparation of Extracts

After preparing the powder of the dried samples by grinding, extraction was performed using the maceration method (with a mixture of ethanol/water 70:30) under agitation and filtration (3×, 24 h each). Extracts were evaporated under reduced pressure at a temperature of less than 40 °C using a rotary evaporator and freeze-dried.

### 3.4. Chromatographic Conditions

Silica gel 60 F2_54_ and silica gel 60 RP-18 F_254_ precoated plates (Merck^®^, Darmstadt, Germany) were used for thin-layer chromatography (TLC) using the following developing systems; S1: ethyl acetate–formic acid–water (82:9:9, *v*/*v*/*v*); S2: water-methanol (0.5:19.5, *v*/*v*); and S3: ethyl acetate–toluene (1:9, *v*/*v*). Different spray reagents, including anisaldehyde–sulfuric acid for the detection of terpenoids, natural product polyethylene glycol reagent (NP/PEG = NEU) for the detection of phenolic acids, and potassium hydroxide (KOH) 5% aqueous solution were used for the detection of anthracene derivatives [68].

Extracts were also analyzed by HPLC using a Waters Alliance 2690 Separations Module (Waters Corporation, Milford, MA, USA) coupled with a Waters 996 photodiode array detector (UV/DAD) (Waters Corporation, MA). The used column was an Atlantis T3, RP-18 end-capped, particle size 5 µm, 150 × 4.6 mm, connected to a pre-column with the same stationary phase. A mixture of water + 0.1% formic acid (solvent A) and acetonitrile (solvent B) was used as the mobile phase. The injection volume was 20 µL with a flow rate of 1mL/min. The following solvent gradient was used: from 0 to 20 min → 95:5 (A:B) to 71:29 (A:B), from 20 to 30 min → 71:29 (A:B) to 67:33 (A:B), from 30 to 35 min → 67:33 (A:B) to 64:36 (A:B), from 35 to 45 min → 64:36 (A:B) to 50:50 (A:B), from 45 to 60 min → 50:50 (A:B) to 0:100 (A:B), from 60 to 65 min 100% (B) and from 65 to 75 min, the initial mobile phase 95:5 (A:B) was used.

Before the analysis, samples were solubilized in water (20 mg/mL) and filtered through a polytetrafluoroethylene syringe filter (0.2 µm). Data were collected and analyzed using Waters Millennium^®^ 32 Chromatography Manager (Waters Corporation, Milford, MA, USA). The chromatogram was monitored and registered on Maxplot wavelength (240–650 nm).

### 3.5. Quantification Assays of the Main Classes of Secondary Metabolites

Total phenolic concentration (TPC) of the crude extracts was determined using the Folin–Ciocalteu method [69] and an increasing gallic acid calibration curve (10–70 µg/mL) was used to obtain the standard equation of Y = 0.0087X + 0.0264, R^2^ = 0.994. Total flavonoid content (TFC) was obtained following the method by Olivera et al., 2008 [70] and catechin concentrations (50–200 µg/mL) were used to obtain a standard curve with equation of Y = 0.0039X + 0.027, R^2^ = 0.993. Total triterpenoid content (TTC) was assessed using the procedure developed by Chang and Lin, 2012 [71] and oleanolic acid concentrations (100–800 µg/mL in methanol) were used to obtain a standard curve with equation of Y = 0.0012X + 0.0849, R^2^ = 0.994. For determination of total condensed tannins (TCTC), the butanol–HCl assay [69] using catechin concentrations (200–2000 µg/mL) was used to obtain a standard curve with equation of Y = 0.0002X + 0.0324, R^2^ = 0.981 and for quantification of total hydrolysable tannins (THTC), the potassium iodate assay [72] with gallic acid concentrations (100–600 µg/mL) was used to obtain a standard curve with equation of Y = 0.001X + 0.054, R2 = 0.977. Total anthraquinones content (TAC) was evaluated according to the method described by Sakulpanich and Gritsanapan, 2008 [73], and rhein concentrations (3–18 µg/mL) were used to obtain a standard curve with the equation of Y = 0.0215X − 0.0016, R^2^ = 0.998.

All the above-mentioned colorimetric techniques were assessed in triplicate for method validation, and a UV-vis spectrophotometer (Hitachi, U–2000) was used. Values were obtained using standard equations (where X was the concentration of standard equivalents expressed as milligrams per gram of dried extract and Y was the measured absorbance). All the obtained data were analyzed statistically by one-way analysis of variance (ANOVA) with *Asphodelus* species as the source of variance. Once both *Asphodelus* species were also collected in two different seasons, the obtained data were then analyzed by ANOVA with the season as the source of variance. The significant value was set for a *p*-value < 0.05.

### 3.6. Genotoxicity/Mutagenicity Evaluation

The Ames test is commonly employed as an initial screening of the genotoxicity potential of herbal substances/preparations because it is rapid, inexpensive, relatively easy to perform, and has been shown to detect relevant genetic changes and most genotoxic carcinogens for rodents and humans [74]. The assessment of mutagenicity in a bacterial reverse gene mutation test (Ames test) is part of the standard genetic toxicology testing battery required by regulatory agencies for the chemical, cosmetic industry, pharmaceutical, and agro-industrial fields to enable the marketing of these products [75]. Regulatory acceptance of the Ames test data often requires the performance of the test according to the Organization for Economic Cooperation and Development (OECD) test guideline 471 [76] and ICH, S2R1 [74].

To assess the mutagenic activity of each extract, the Ames test was performed using the method described by Maron and Ames, 1983 [67] following the OECD (471) [76] and ICH S2 (R1) guidelines [74]. Briefly, extracts dissolved in DMSO (up to 30%), which also served as the negative control, and concentrations of 250, 625, 1250, 2500, 3750, and 5000 µg/plate were tested for mutagenicity using five *Salmonella enterica* serovar Typhimurium tester strains (TA98, TA100, TA102, TA1535 and TA1537) in plate incorporation assay without metabolic activation. Sodium azide, 2-nitrofluorene, tert-butyl-hydroperoxide, and 9-aminoacridine were used as positive controls for TA100 and TA1535, TA98, TA102, and TA1537, respectively. Assays were performed in triplicate, and manual counting of His+ revertant colonies for each concentration was performed after 48 h incubation at 37 °C. Results are expressed as the mean number of revertant colonies with standard deviations (means ± SD) for each strain in different concentrations.

## 4. Conclusions

In conclusion, the obtained results of our study allowed the establishment of the morphological characteristics needed for the identification of *A. bento-rainhae* and *A. macrocarpus* root tubers as raw materials for pharmaceutical use. Considering the obtained results of the Ames tests, the extracts tested up to 5.0 mg/plate (maximum test concentration recommended by the OECD guidelines for testing chemicals) did not induce an increase in the number of revertants per plate in any of the tested bacterial strains; therefore, under the conditions of our study, the extract did not show genotoxic potential. These data, together with the phytochemical profile determination and quantification of the main constituents present in each of these medicinal plants, provide relevant information for inclusion in their quality monographs, and more importantly, draw attention to the need for conservation action and eventually prevent their extinction.

## Figures and Tables

**Table 1:** Principal morphological and anatomical features of *A. bento-rainhae* and *A. macrocarpus* root tubers.

Anatomical Characteristic	AbR			AmR		
	Min–Max	Mean	±SD	Min–Max	Mean	±SD
Root length (cm)	2–5	3.5	0.7	6–13	8.7	2.2
Root diameter (cm)	0.7–1.6	1	0.2	1.2–1.7	1.4	0.2
Velamen (numbers of cell layers)	4–5	4	0.25	4–7	5	0.54
Cortex (numbers of cell layers)	17–24	21	3.1	21–37	29	4.7
Idioblast cell width (μm)	58.3–62.5	134	2.9	60.7–114.6	150	27.6
Protoxylem wall thickness (μm)	4.2–5	4.5	0.4	4.1–6	4.6	0.3
Protoxylem diameter (μm)	20.8–25	22.9	2.95	8.33–61	36.1	35.8
Metaxylem wall thickness (μm)	8.3–13.9	10.4	1.7	10.8–14.6	12.4	1.6
Metaxylem diameter (μm)	50–99.6	70.3	13.1	52–101.8	80.7	19.0
Pith cell diameter (μm)	73.2–121.9	93	5.6	94.4–140.7	114	4.6
Raphids length (μm)	20.8–62.5	37.2	14.2	78–114.3	87.7	15.3

Abbreviations: AbR, *A. bento-rainhae* root; AmR, *A. macrocarpus* root; Min, minimum; Max, maximum; SD, standard deviation.

**Table 2:** Quantification of principal chemical classes of *A. bento-rainhae* and *A. macrocarpus* root tuber extracts.

Assays	AbRa	AbRb	AmRa	AmRb
	Mean ± SD	Mean ± SD	Mean ± SD	Mean ± SD
**TPC**				
(mg GAE/g dried extract)	20.36 ± 4.2	26.45 ± 7.52	29.14 ± 9.32	27.35 ± 8.13
(mg GAE/g dried Root)	10.94 ± 2.26	13.76 ± 3.91	12.76 ± 4.08	10.12 ± 3.01
**TFC**				
(mg CAE/g dried extract)	10.55 ± 1.17	16.71 ± 1.12 *	18.90 ± 0.26	17.70 ± 0.24
(mg CAE/g dried Root)	5.67 ± 0.63	8.69 ± 0.58	8.28 ± 0.11	6.55 ± 0.09
**TTC**				
(mg OAE/g dried extract)	173.88 ± 29.82	172.11 ± 19.20	180.55 ± 10.57	154.36 ± 20.53
(mg OAE/g dried Root)	93.46 ± 16.03	89.50 ± 9.99	79.08 ± 4.63	57.11 ± 7.60
**TAC**				
(mg RhE/g dried extract)	2.43 ± 0.17	3.21 ± 0.21 *	3.38 ± 0.26 *	2.68 ± 0.19
(mg RhE/g dried Root)	1.31 ± 0.12	1.67 ± 0.16	1.48 ± 0.14	0.99 ± 0.09
**TCTC**				
(mg CAE/g dried extract)	93.80 ± 9.39	128.64 ± 14.05 *	88.08 ± 7.83	108.35 ± 20.37
(mg CAE/g dried Root)	50.42 ± 20.76	66.89 ± 7.30	38.58 ± 3.43	40.09 ± 7.54
**THTC**				
(mg GAE/g dried extract)	21.91 ± 7.43	32.73 ± 8.61	25.81 ± 7.25	28.09 ± 6.16
(mg GAE/g dried Root)	11.78 ± 4.91	17.02 ± 4.48	11.31 ± 3.17	10.39 ± 2.28

Abbreviations: AbRa, *A. bento-rainhae* root 1st collection; AbRb, *A. bento-rainhae* root 2nd collection; AmRa, *A. macrocarpus* root 1st collection; AmRb, *A. macrocarpus* root 2nd collection; SD, standard deviation; TPC, total phenolic content; TFC, total flavonoid content; TTC, total triterpenoid content; TAC, total anthraquinones content; TCTC, total condensed tannin content; THTC, total hydrolysable tannin content; GAE, gallic acid equivalents; CAE, catechin equivalents; OAE, oleanolic acid equivalents; RhE, rhein equivalents. * Significantly higher content (*p*-value < 0.05) when compared between different seasons of collection of the same species.

**Table 3:** Mutagenicity of root extracts in the bacterial reverse mutation test (Ames test).

Extracts	Plate Incorporation Test without Metabolic Activation									
	TA98		TA100		TA102		TA1535		TA1537	
	Mean	±SD	Mean	±SD	Mean	±SD	Mean	±SD	Mean	±SD
**AbRb** **µg/plate**										
250	16	3.1	174	7.8	365	21.4	19	4.6	10	1.2
625	21	2.5	158	3.1	334	14.6	20	3.1	11	2.3
1250	23	3.1	164	9.9	354	16.3	25	2.5	10	1.7
2500	23	1.5	177	22.1	363	8.9	20	0.6	10	2.1
3750	20	1	164	2.3	392	41.3	19	3.1	10	2.1
5000	23	3.6	183	17.4	365	19.8	17	1.7	10	3.2
**AmRb** **µg/plate**										
250	21	4.6	177	13.1	347	9	26	5.6	9	1.2
625	18	0.6	158	8.2	354	9.5	23	3.5	9	2.1
1250	22	6.1	179	17	379	29.5	17	1.2	11	2.1
2500	23	5.3	177	7.6	397	22.6	21	1.2	10	2
3750	21	5.2	179	12.5	394	10	19	0.6	11	2.1
5000	22	1	166	16.1	395	28.8	17	1.5	9	1
**NR**	19	1.5	156	16.7	320	3.5	21	2.5	7	1
PR *	487.7	30.2	1048	43.2	881	26.2	827.3	13.1	1354	4.5

Abbreviations: AbRb, *A. bento-rainhae* root 2nd collection extract; AmRb, *A. macrocarpus* root 2nd collection extract; SD, standard deviation; NR, negative reference; PR, positive reference. * Positive control references: TA98, 2-nitrofluorene (5 μg/plate); TA100, sodium azide (1.5 μg/plate); TA102 *tert*-butyl-hydroperoxide (50 μg/plate); TA1535, sodium azide (1.5 μg/plate) and TA1537, 9-aminoacridine (100 μg/plate).

## Data Availability

Not applicable.
